# Meta-proteomics of rumen microbiota indicates niche compartmentalisation and functional dominance in a limited number of metabolic pathways between abundant bacteria

**DOI:** 10.1038/s41598-018-28827-7

**Published:** 2018-07-12

**Authors:** E. H. Hart, C. J. Creevey, T. Hitch, A. H. Kingston-Smith

**Affiliations:** Institute of Biological, Environmental and Rural Sciences (IBERS), Aberystwyth University, Penglais, Aberystwyth SY23 3FG UK

## Abstract

The rumen is a complex ecosystem. It is the primary site for microbial fermentation of ingested feed allowing conversion of a low nutritional feed source into high quality meat and milk products. However, digestive inefficiencies lead to production of high amounts of environmental pollutants; methane and nitrogenous waste. These inefficiencies could be overcome by development of forages which better match the requirements of the rumen microbial population. Although challenging, the application of meta-proteomics has potential for a more complete understanding of the rumen ecosystem than sequencing approaches alone. Here, we have implemented a meta-proteomic approach to determine the association between taxonomies of microbial sources of the most abundant proteins in the rumens of forage-fed dairy cows, with taxonomic abundances typical of those previously described by metagenomics. Reproducible proteome profiles were generated from rumen samples. The most highly abundant taxonomic phyla in the proteome were *Bacteriodetes*, *Firmicutes* and *Proteobacteria*, which corresponded with the most abundant taxonomic phyla determined from 16S rRNA studies. Meta-proteome data indicated differentiation between metabolic pathways of the most abundant phyla, which is in agreement with the concept of diversified niches within the rumen microbiota.

## Introduction

The rumen plays host to a complex microbiome consisting of over seven thousand species from protozoa, archaea, bacteria and fungi and is the primary site for microbial fermentation of ingested feed in ruminants^1^. This symbiotic relationship allows the host to utilise the nutrients in otherwise indigestible lignocellulose plant material^2^. However, inefficiencies in this system result in lost energy and negative impacts including production of greenhouse gases and leaching of nitrogenous pollutants into the environment^3^. Opportunities exist to mitigate these impacts and increase efficiency by modification of rumen function via improved feeds, however this requires a greater understanding of the rumen microbiome at a systems-level^4^.

To date culturing techniques and more recently ‘omics technologies have been directed towards enhancing knowledge of the diversity, structure and function of the rumen microbial community^4–10^. Metagenomic sequencing data is frequently used to assess the functional potential of the rumen microbiome and recently meta-transcriptomics has increasingly been used to predict function^11^. However, translation does not always directly follow gene expression patterns and so functionality needs to be confirmed at the protein level and through understanding the effect of post-translational modifications. Proteome evaluation is not a trivial task in complex environments such as the rumen and thus the information from the rumen meta-proteome is much poorer than that generated from meta-genomic and meta-transcriptomic analyses.

Meta-proteomics has been described as the entire protein complement of the microbiota in an environment at a given point in time^12^. Aqueous, terrestrial and eukaryotic (humans, mice, termites and plants) ecosystems have been the focus of most meta-proteomic analyses to date^13^. In complex samples, such as the rumen, proteomic analyses are incredibly challenging^14^, not least because of the presence of interfering compounds such as humic substances, which are difficult to remove from the sample and can bind to proteins and cause structural modification^15–17^. If these issues could be solved, analysis of the meta-proteome of the rumen microbial community would reveal details about microbial community activity, structure, function and metabolic pathway transformations that are presently lacking.

The aims of this study were (1) To use a meta-proteomic approach to analyse the rumen system to determine if the most abundant proteins present matched those predicted by meta-transcriptomic data. (2) Use this information to identify the most translationally active organisms in the rumen microbiome. This information will aid in the further understanding of the active microbial metabolic pathways in the rumen of cows fed fresh forage. Previously, Snelling and Wallace^14^ reported inconsistent proteomic gel images from rumen samples, which limited interpretation. The methodology described here produced reproducible proteome profiles of a quality suitable for more detailed investigations. By comparison of our data with that derived from 16S rRNA profiling from comparable data sets^10^, it was possible to compare the taxonomic abundances derived from protein family identification to rumen microbe transcription profiles^18^, to enable the exploration of the abundance/functional relationship underpinning rumen resilience^19^.

## Results

### Reproducibility of the rumen metaproteome

**Figure 1:**
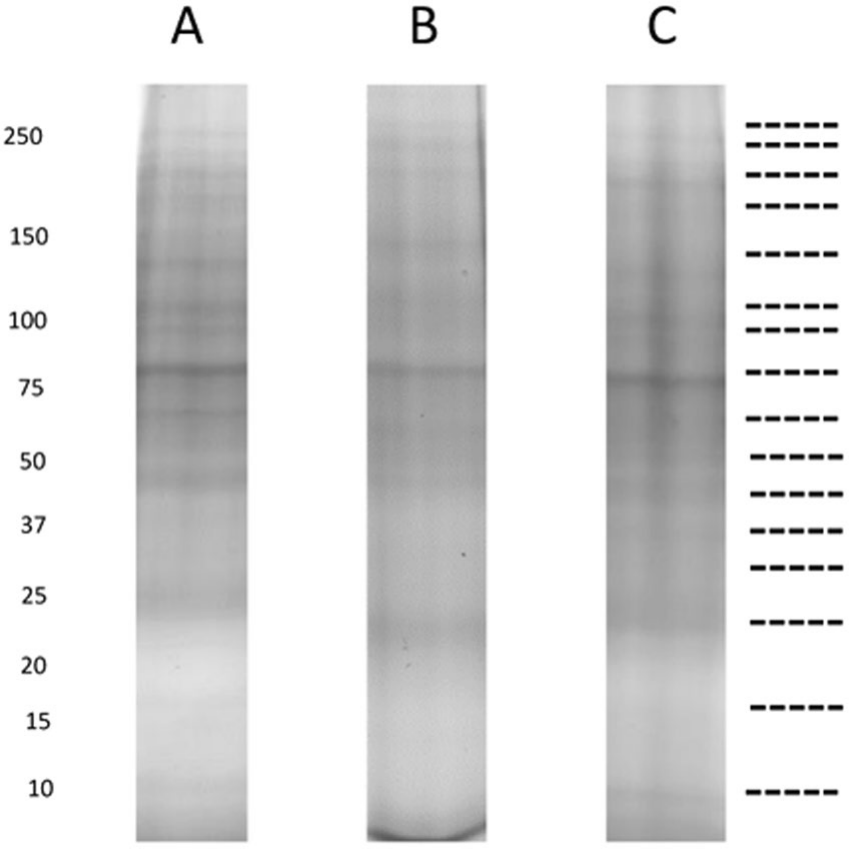
SDS-PAGE (12.5% T, 3.3% C) images of Cow 1 (**A**) cow 2 (**B**) and cow 3 (**C**) rumen proteins, showing distinct bands at similar molecular weights between cows. Dotted lines indicate where the lanes were cut to generate the gel slices for MS analysis.

Preliminary investigations showed that previously published methodologies for extraction of protein for proteomic analysis such as TCA/acetone precipitation^20,21^, the direct use of detergents^22–24^, phenol/methanol extraction^25,26^ or a combination of methods^27^ did not generate the purity required for proteomic analysis of protein extracts from rumen samples (data not shown). In contrast, an extraction methodology involving serial washing using a 0.9% NaCl solution (described by Wilmes and Bond^12,28^ as being effective in other systems in removing large quantities of interfering substances from the sample), a separation step using a 40% Percoll solution (Supplementary Fig. S1) and subsequent urea/detergent based protein extraction (as described in Materials and Methods) enabled multiple, distinct bands to be distinguished in rumen samples from cows by one dimensional SDS-PAGE (Fig. 1).

**Figure 2:**
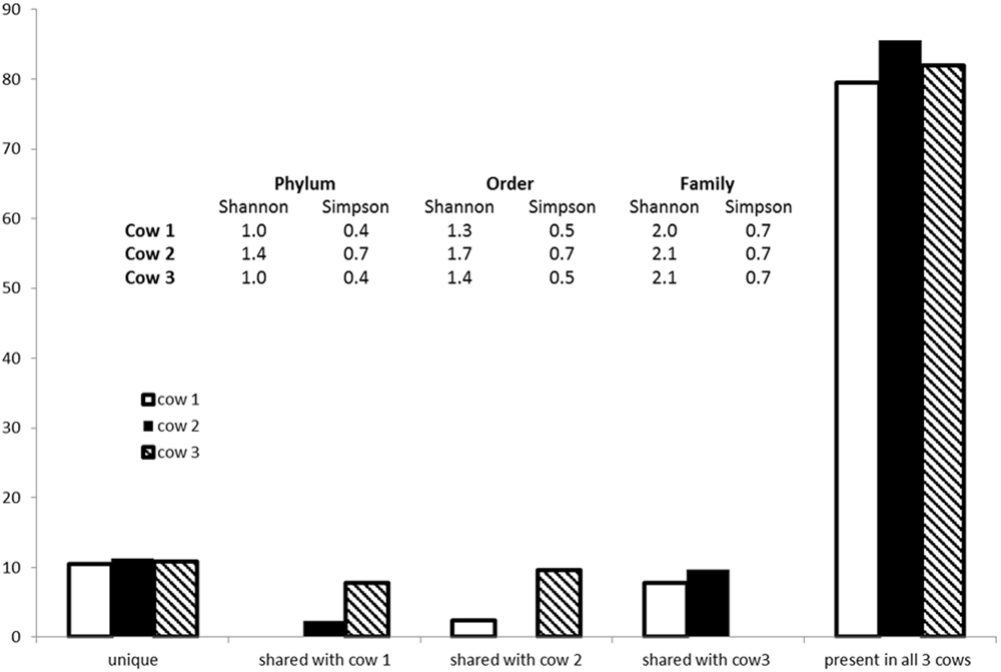
Percentage of overlap of protein families shared and unique between each cow replicate and table of the Shannon and Simpson diversity index comparing each cow in terms of taxonomy based on Phylum, Order and Family levels.

The SDS-PAGE protein profiles (Fig. 1) produced for each cow replicate (n = 3) were subjected to MS/MS analysis after the entire lane was divided into sections as indicated in Fig. 1 so that the proteins contained in the entire lane were sequenced. These sections were uneven to account for differential band density and so maximise detection of low abundance proteins from poorly staining regions of the gel. Nevertheless it was noted that most information was derived from those gel sections containing recognisable bands. Database interrogation and peptide sequence filtering was undertaken as per parameters outlined in Materials and Methods. A total of 167,506 putative peptides were generated across all the samples of which 93, 957 (56%) had a match to the non-redundant NCBI database (all replicates combined). Individually, this was comprised of 32,845 (55% total peptides), 28,632 (56% total peptides) 32,480 (55% total peptides) peptides from cows 1, 2 and 3 respectively. Protein identifications and taxonomic classifications were similar between cow replicates, showing reproducibility of the methodology for sample preparation. Over 80% of protein families detected were common to all three cow replicates with only 10% unique to each cow. Less than 10% of protein families were present in only two out of the three cow replicates (Fig. 2). Differences in protein diversity between cow replicates were very low at phylum, order and family level as determined from Shannon and Simpson diversity comparisons (Fig. 2).

### Distribution of abundant organisms

**Figure 3:**
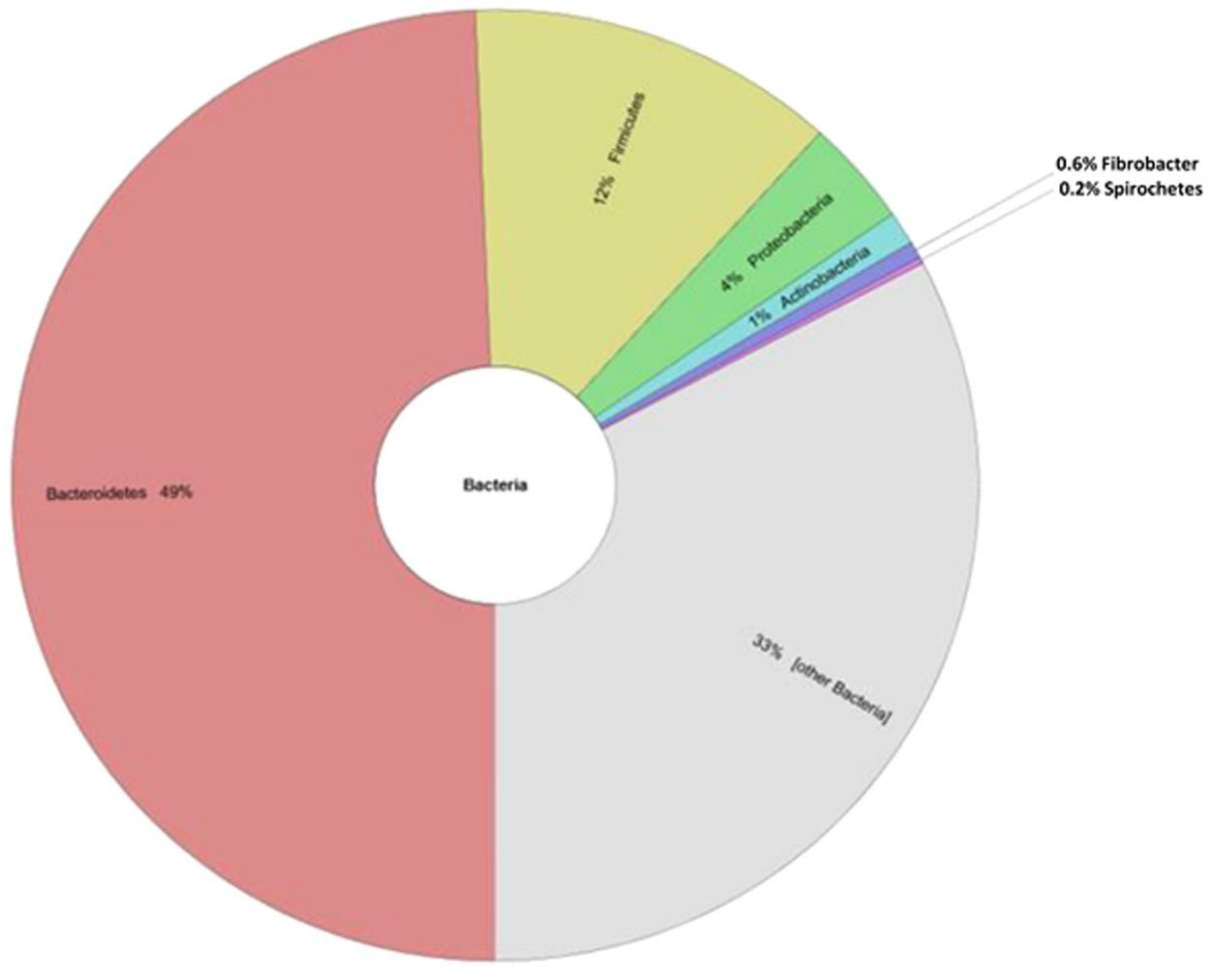
Krona plot of the percentage abundance of the most common phyla determined from the domain bacteria (representing the most abundant domain determined from analyses). The top three categories of the most abundant phyla shown here are Bacteriodetes, Firmicutes and Proteobacteria.

No significant differences were observed in the overall abundance and distribution of taxonomic profiles between replicates (Fig. 2). Within the rumen metaproteome the abundance of bacterial proteins was found to be significantly greater than that of fungal, plant or protozoal proteins in the samples assessed using this methodology. Of the taxa determined by Unipept analysis^29^, bacteria represented 77% of all taxa with the phylum Bacteroidetes as the most abundant (49% abundance) followed by Firmicutes (12% abundance) and Proteobacteria (4% abundance; Fig. 3). To determine whether these differences were robust to the database analysis performed, analyses were also performed using Metaproteome analyser^30^. This analysis confirmed these three bacterial phyla as the most abundant, although there were some slight differences in relative percentage abundances as compared with analysis by UniPept with Bacteroidetes, Firmicutes and Proteobacteria comprising 67%, 16% and 5% of taxonomic abundances respectively.

To determine if the data obtained from this metaproteomic study could be identified in organisms from the rumen, a comparison of our rumen metaproteome was made against the Hungate 1000 genome database (of which 406 genomes were available)^10^. We identified hits within 346 of the Hungate genomes. To identify if the taxonomic profiles identified from the metaproteome were representative of those known from the rumen, we also carried out a comparison with previously published 16S rRNA datasets^10^. This demonstrated 88% (cows 1 and 3) and 75% (cow 2) of the phyla identified from the metaproteome were known to exist in the 16S rRNA datasets. When comparing further taxonomic rankings of the metaproteome data to the 16S rRNA datasets^10^, 76%, 52% and 61% (cows 1, 2 and 3 respectively) matched at the order level and at family level matches were 67% for cow 1, 48% for cow 2 and 61% for cow 3 showing a tendency towards a slight decrease in the taxonomic correlations as taxonomy becomes more specific.

### Functional characterisation of the rumen meta-proteome

**Figure 4:**
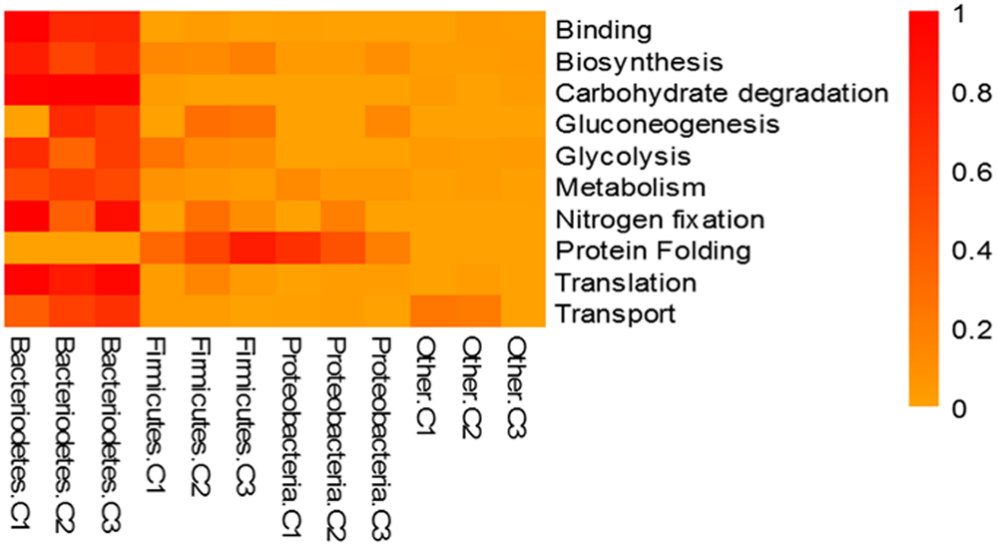
Heat map showing percentage abundance of top 25 protein family summary functions against the top phyla present for cow 1 (C1), cow 2 (C2) and cow 3 (C3). Please see supplementary Table 2 for further information.

**Figure 5:**
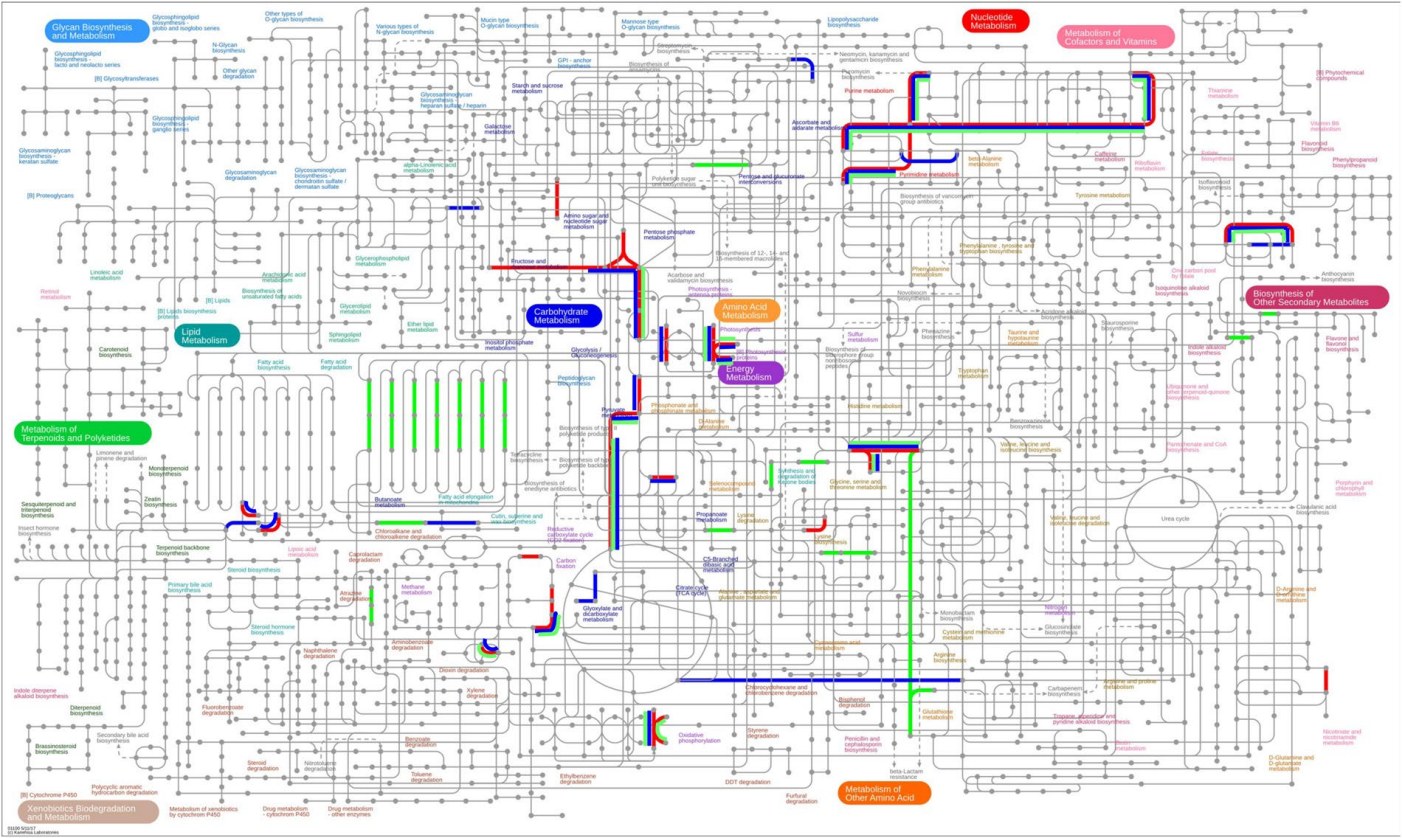
KEGG map (www.kegg.jp) showing main metabolic pathways generated from each cow replicate. Red depicts main pathways represented by cow 1, blue from cow 2 and green from cow 3.

**Figure 6:**
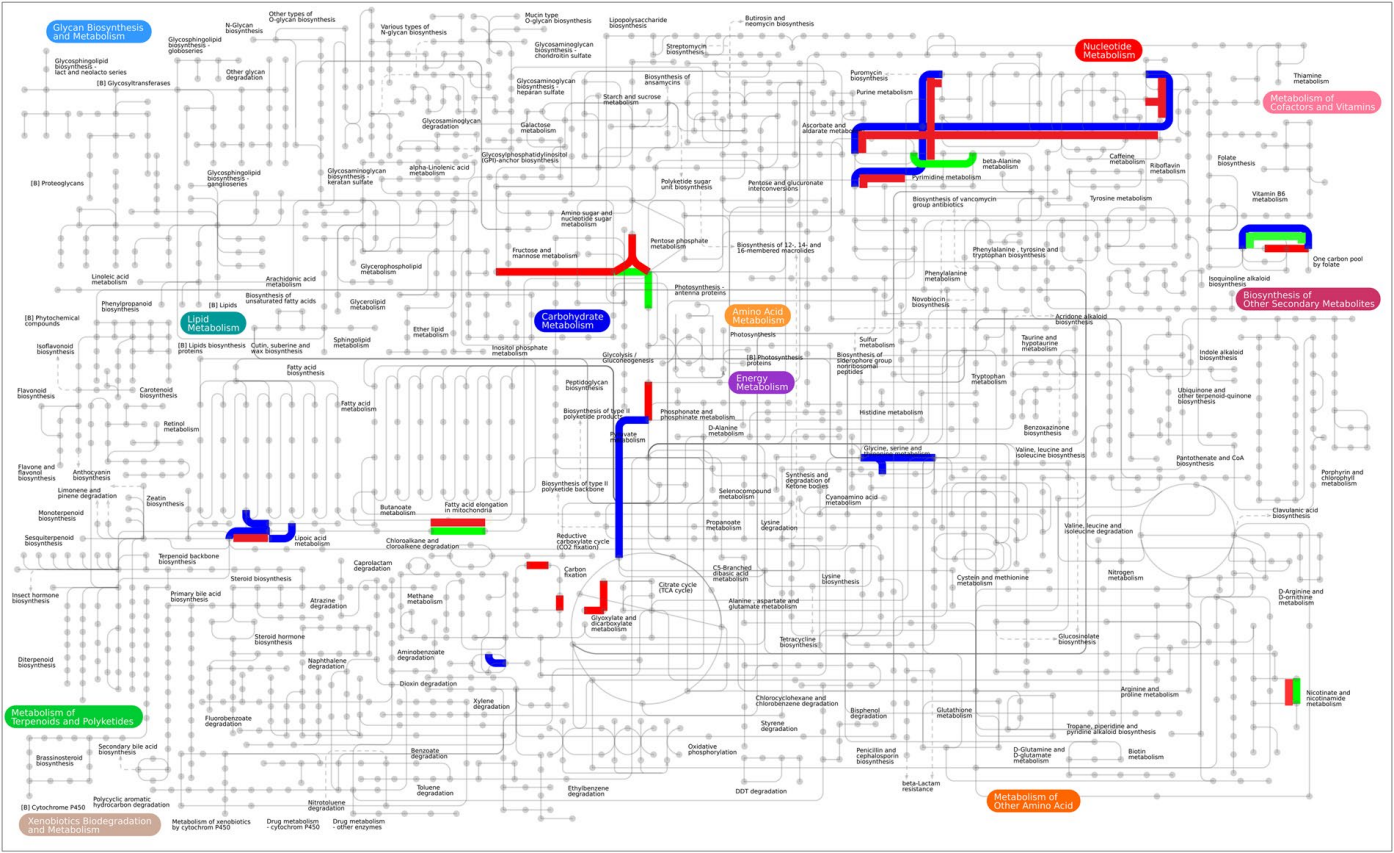
KEGG map (www.kegg.jp) showing the main metabolic pathways belonging to the dominant taxa observed in this study (Fig. 4). The three main phyla are represented as follows, Bacteriodetes shown in blue, Proteobacteria in green, Firmicutes in red. Differentiation of pathways can be observed between phyla.

Protein families were categorised into functional components per biological processes, molecular functions and cellular location. Gene ontology analysis showed that most of the highly abundant proteins were categorised into ten biological processes which varied little amongst cow replicates. Glycolysis, electron transport and carbohydrate metabolism were predominant pathways with major molecular functions relating to oxidoreductase, isomerase, kinase and binding activities identified (Table 1). The subcellular locations of the top 25 protein families were determined to be present in the cytoplasm for 60% of the highly abundant proteins with the remainder determined at 16% for membrane proteins, 20% and 4% for cytosolic and nucleus bound proteins respectively (Supplementary Data Table 1). At the phylum level the distribution of the top 25 protein family summary functions revealed that Bacteriodetes were most highly represented for nearly all the major protein functions determined, apart from protein folding (Fig. 4). Mapping the 25 most abundant protein families from each cow replicate (Supplementary Data Table 1 and Fig. 5) using iPATH (interactive pathways explorer 2^31,32^) from KEGG identifiers^33^ revealed that many pathways from all 3 cows were related to carbohydrate metabolism, nucleotide metabolism, pyruvate metabolism, amino acid metabolism, glycine, serine and threonine metabolism, oxidative phosphorylation and in the biosynthesis of other secondary metabolites (Fig. 5). Analysis of KEGG pathways information showed over 40% of pathways identified were shared between all cows (Supplementary Data Fig. 2), with less than 30% of pathways unique to each cow. Pathway mapping was also carried out on the top most abundant phyla (65%) observed from this study (Fig. 4), showing mapping differences between Bacteriodetes, Proteobacteria, Firmicutes (Fig. 6). The main pathways highlighted for Bacteriodetes were involved in those designated as nucleotide metabolism, carbon metabolism, carbohydrate and lipid metabolism and lipoic acid metabolism, which could be further broken down into fatty acid metabolism, valine, leucine and isoleucine degradation, glycolysis, gluconeogenesis, TCA cycle, and pyruvate metabolism. Pathways identified in Firmicutes were nucleotide, carbon metabolism and carbohydrate and lipid metabolism, which were further divided into purine metabolism, pyrimidine metabolism, glycolysis, gluconeogenesis, pentose phosphate pathways, fructose and mannose metabolism, terpenoid synthesis and nitrogen metabolism. Proteobacteria were involved in nucleotide metabolism, carbon metabolism and carbohydrate and lipid metabolism, which could be further subdivided into pyrimidine metabolism, glycine, serine and threonine metabolism, methane metabolism and nitrogen metabolism.

**Table 1:** The distribution of the top 25 protein families for each cow replicate and their percentage abundance.

Cow 1			Cow 2			Cow 3		
Top 25 Protein Families	Summary Function	Percentage abundance	Top 25 Protein Families	Summary Function	Percentage abundance	Top 25 Protein Families	Summary Function	Percentage abundance
elongation factor Tu	Biosynthesis	44.70	Serum albumin	Transport	25.75	elongation factor Tu	Biosynthesis	31.82
Serum albumin	Transport	10.76	glyceraldehyde-3-phosphate dehydrogenase	Glycolysis	18.01	glyceraldehyde-3-phosphate dehydrogenase	Glycolysis	11.61
pyruvate, phosphate dikinase	Transport	7.96	pyruvate, phosphate dikinase	Transport	9.33	50S ribosomal protein	Translation	11.35
glutamate dehydrogenase	Biosynthesis	7.42	elongation factor Tu	Biosynthesis	7.05	30S ribosomal protein S1	Biosynthesis	8.53
phosphoglycerate kinase	Glycolysis	4.47	50S ribosomal protein	Translation	6.88	glutamate dehydrogenase	Biosynthesis	7.29
50S ribosomal protein	Translation	4.44	30S ribosomal protein S1	Biosynthesis	3.14	hypothetical protein	Metabolism	4.09
glyceraldehyde-3-phosphate dehydrogenase	Glycolysis	3.57	triose-phosphate isomerase	Gluconeogenesis	2.71	triose-phosphate isomerase	Gluconeogenesis	2.35
phosphoenolpyruvate synthase	Metabolism	2.90	thioredoxin	Protein Folding	2.06	thioredoxin	Protein Folding	2.09
30S ribosomal protein S1	Biosynthesis	2.62	succinate dehydrogenase	Metabolism	1.93	phosphoglycerate kinase	Glycolysis	1.83
ATP synthase subunit beta	Transport	1.72	nitrogen-fixing protein NifU	Nitrogen fixation	1.72	succinate dehydrogenase	Metablolism	1.77
thioredoxin	Protein Folding	1.23	hypothetical protein	Metabolism	1.63	nitrogen-fixing protein NifU	Nitrogen fixation	1.47
hypothetical protein	Metabolism	1.10	transketolase	Metabolism	1.55	pyruvate, phosphate dikinase	Transport	1.41
molecular chaperone DnaK	Binding	1.00	fructose-bisphosphate aldolase	Glycolysis	1.33	transketolase	Metabolism	1.18
fucose isomerase	Carbohydrate degradation	0.77	DNA-directed RNA polymerase, alpha subunit	Binding	1.25	molecular chaperone DnaK	Binding	1.05
rubrerythrin	Binding	0.69	phosphoenolpyruvate carboxykinase (ATP)	Gluconeogenesis	1.25	phosphoenolpyruvate carboxykinase (ATP)	Gluconeogenesis	1.05
succinate dehydrogenase	Metabolism	0.69	pyruvate ferredoxin (flavodoxin) oxidoreductase	Transport	1.20	ribosomal proteins	Biosynthesis	0.98
DNA-directed RNA polymerase subunit beta	Binding	0.56	racemase	Metabolism	1.20	pyruvate ferredoxin (flavodoxin) oxidoreductase	Transport	0.95
ribosomal proteins	Biosynthesis	0.44	molecular chaperone DnaK	Binding	1.03	DNA-directed RNA polymerase, alpha subunit	Binding	0.69
nitrogen-fixing protein NifU	Nitrogen fixation	0.28	ATP synthase subunit beta	Transport	0.95	fucose isomerase	Carbohydrate degradation	0.69
diphosphate–fructose-6-phosphate 1-phosphotransferase	Glycolysis	0.26	pyruvate synthase	Transport	0.90	rubrerythrin	Binding	0.65
phosphoglucomutase	Metabolism	0.26	phosphoglucomutase	Glycolysis	0.77	methylmalonyl-CoA mutase	Metabolism	0.49
racemase	Metabolism	0.23	ribosomal proteins	Biosynthesis	0.77	energy transducer TonB	Transport	0.46
transketolase	Metabolism	0.23	rubrerythrin	Binding	0.77	phosphoglucomutase	Glycolysis	0.43
pyruvate ferredoxin (flavodoxin) oxidoreductase	Transport	0.21	fucose isomerase	Carbohydrate degradation	0.60	fructose-bisphosphate aldolase	Glycolysis	0.39
transcription termination factor NusA	Binding	0.21	serine hydroxymethyltransferase	Metabolism	0.52	pyruvate synthase	Transport	0.39

### Comparison of meta-proteome analyses to meta-transcript abundance

**Figure 7:**
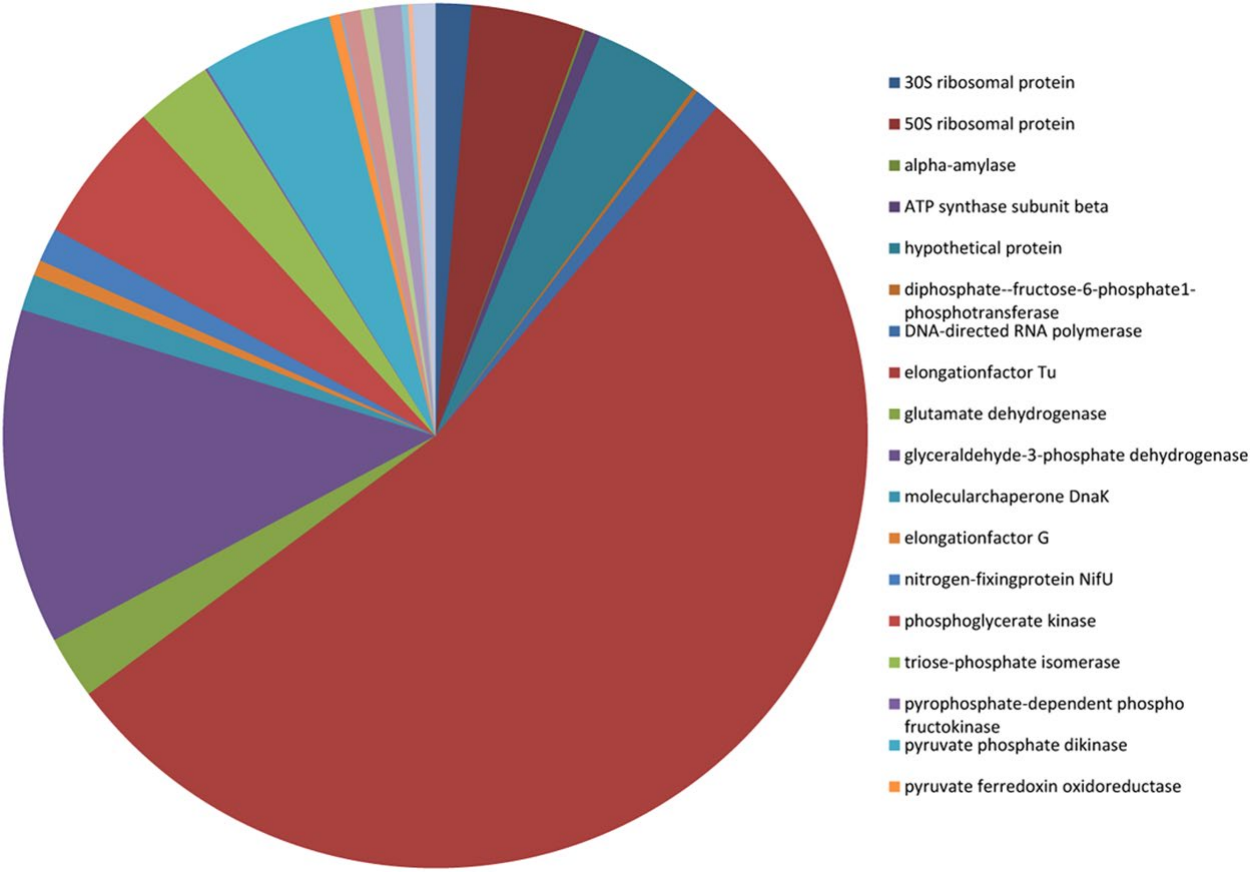
The most abundant protein families present from the meta-proteomic data and their percentage distribution amongst the transcriptome data set from Shi *et al*.^18^ to matched contigs.

A comparison was conducted to determine whether the most highly abundant peptides expressed from proteomic analyses correlated with the most highly expressed transcripts from meta-transcriptomic datasets from the rumen using an extremely large rumen meta-transcriptome data set^18^. Although 80% of the transcriptome from Shi *et al*.^18^ aligned with the metagenome assembly^10^, the number of genes expressed was small compared to the entirety of the genes represented in the metagenome (0.89% of the metagenome). Of those expressed genes, there were matches with 71% of the metaproteome. The most abundant protein families present in the metaproteomic data and their percentage distribution amongst matched contigs in the transcriptome data set are shown in Fig. 7, where elongation factor Tu, glyceraldehyde-3-phosphate dehydrogenase and phosphoglycerate kinase were the most abundant matches to the transcriptomic data.

## Discussion

The rumen microbial consortium is composed of thousands of species of bacteria, methanogenic archaea, protozoa, fungi and viruses with bacteria being the most abundant micro-organisms in the rumen reaching up to 10^11^ cells/ml rumen fluid^34^. The extensive microbial diversity of the rumen^9,35^ confers resilience to the host and the ability to adapt to different dietary materials^36^. However, animal-to-animal variation in rumen bacterial communities can impact the response of individuals to changes in diet^37^. Indeed, it is thought that diet is one of the major drivers of taxonomic composition in the rumen microbiome^37–39^. Understanding how changes in microbial diversity affect the function of the rumen or enable sustained feed utilisation is a current challenge for ruminant science.

Predictions based on gene sequences from culture-independent and metagenomic techniques have shown that there is considerable redundancy in the rumen ecosystem^40^. In particular, the potential for expression of key enzymes by multiple species makes determining the relationship between microbial abundance and functionality far from straightforward. In this work, we used a meta-proteomic approach to assess the relationship between the abundance of rumen proteins, their source organisms and typical patterns of gene expression from meta-transcriptomic data.

Brulc *et al*.^37^ determined that the distribution of rumen phylotypes falls predominantly into three main categories; Bacteriodetes, Firmicutes and Proteobacteria, regardless of the microbiome analysed. Within this, Henderson *et al*.^39^, considered there to be a “core bacterial microbiome”, in which Bacteriodetes and Firmicutes predominate. Supporting this, the average composition of the rumen bacterial community from metagenomic data was found to be 50% Bacteriodetes, 43% Firmicutes and 5.4% Proteobacteria^41^. By reference to published metagenomes, it was possible to similarly characterise the trend of protein diversity of the rumen by their predicted species of origin. We found that the metaproteome was in agreement with the taxonomic profiles from metagenomic and 16S rRNA profiles^10,41^ with Bacteriodetes, Firmicutes and Proteobacteria representing most highly abundant phyla. The protein extraction protocol developed here is effective and reproducible for extraction of bacterial proteins from rumen samples. Finally, we showed high replicability between the cows sampled both taxonomically and in protein sequence abundance.

The rumen microbiota gain energy from glucose units derived from the degradation of fibre which is used to drive microbial protein synthesis and proliferation. Fibre degradation is considered to be due to the activity of a consortium of fibrolytic bacteria^42^. Once thought to be abundant in the rumen, *Fibrobacter succinogens*, typically only represents 1–3% of total rumen bacteria^9,42,43^ and has been found to be missing entirely in up to half of samples analysed in some studies^41^. Similarly, relatively few peptides in our data could be identified as originating from *F*. *succinogenes* and the functions of those detected were primarily involved in protein biosynthesis. The lack of fibrolytic activity in these samples is surprising. Firstly this could be a result of lack of translation. For example glycohydrolase activity has been shown to be an inducible part of colonisation^44^ and for these studies rumen fluid was collected from animals which had been fasting overnight. Greater presence of glycohydrolases would be predicted from samples taken during feeding. Secondly, we have described here improvements to the protocol for extraction of proteins from rumen bacteria. It is recognised that detection of cell-free extracellular proteins of bacterial origin (which could include fibrolytic activity) will require further modification to separate proteins from those contaminants (eg humic acids) which are abundant in the rumen liquor.

While functional analysis of the rumen meta-proteome identified proteins involved in diverse metabolic processes, unsurprisingly they originated from only a few bacterial phyla representing those most abundant in the rumen. The most abundant protein functions observed were associated with ontologies representing core metabolic pathways such as, glycolysis, gluconeogenesis, protein biosynthesis and electron transport (Table 1). While these activities were identified in more than one phylum (Fig. 4), the largest proportion were from Bacteroidetes suggesting a central role of this phylum in microbial metabolism and reflecting phylogenetic dominance. Snelling and Wallace^14^ also found that more proteins belonged to Bacteroidetes than Firmicutes or Proteobacteria.

The most highly abundant protein family found belonged to elongation factor Tu, which accounts for 5 to 10% of total cell protein^45^. These proteins, along with 30S and 50S ribosomal proteins are important in protein biosynthesis, where the GTP-dependent binding of aminoacyl-tRNA to the A-site of ribosomes is promoted. Glycolysis and the citric acid cycle provide sources of energy within the rumen^46^. In this study, multiple components of the glycolytic pathway (glucose to pyruvate conversion) were detected within the rumen metaproteome with glyceraldehyde-3-phosphate dehydrogenase found to be the most abundant protein contributing to this biological process in the rumen all cows. This enzyme is involved in the first step in the glycolytic pathway catalysing the reversible oxidative phosphorylation of D-glyceraldehyde-3-phosphate to 1,3-bisphospho-D-glycerate in the presence of NAD+ and phosphate. The most common phyla contributing to this process was the Firmicutes, with the families Ruminococcaeae and Lachnospiracaea dominating. However, other contributing phyla included Bacteriodetes, Proteobacteria, Spirochetes and Fibrobacteres. Given its core role, proteins associated with glycolysis are likely to be present in all phyla. Therefore we consider that the differential detection most likely relates to differential abundance of the phyla within the rumen microbiome or to a lack of annotated sequence data. In the second step of the glycolysis pathway dihydroxyacetone phosphate (DHAP) is converted to D-glyceraldehyde-3-phosphate and phosphoglycerate kinase by the triosphosphate isomerase, which was found to be highly abundant in the metaproteome. The same was observed with fructose bisphosphate aldolase involved in step 4 of the glycolytic pathway, showing an abundance of proteins relating to glycolysis distributed within the phylum Bacteriodetes, Firmicutes and Proteobacteria. The detection of multiple proteins from the same pathway increases the confidence that a particular pathway is represented *in vivo*.

Electron transport featured highly in the top 25 most highly abundant protein families from the metaproteome. This included ATP synthase, thioredoxin, rubrythrin and pyruvate synthase, the latter of which is required for the transfer of electrons from pyruvate to ferredoxin for the generation of VFA by the rumen microbiome. Pyruvate can be the central intermediary metabolite in rumen metabolism and is the branch point where pathways diverge to form various fermentation products including the major VFAs namely acetate, propionate and butyrate^1^. Not unsurprisingly pyruvate: ferredoxin (flavodoxin) oxidoreductase was also amongst the most highly abundant proteins identified. This is linked to the pyruvate pathway and required by intestinal anaerobes for the oxidation of NADH and the production of VFA that are absorbed into the animal’s bloodstream and used for energy generation.

Phosphoenolpyruvate carboxykinase was detected in the rumen metaproteome of two of the three cows. These genes have been reported to have a relatively high expression in cow rumen datasets^47^ and is thought to be one of the enzymes involved in the fermentation of cellulose to succinate. Phosphoenolpyruvate carboxykinase catalyses the conversion of oxaloacetate (OAA) to phosphoenolpyruvate (PEP) through direct phosphoryl transfer between the nucleoside triphosphate and OAA, as part of the gluconeogenesis pathway. However, this reaction is reversible and may operate in the direction of pyruvate formation and so may contribute to availability of substrates for fermentation. The catalysis of the phosphorylation of pyruvate is driven by pyruvate phosphate dikinase. In this study, the peptides associated with this central function were determined to belong to the phylum Firmicutes, particularly from the families Lachnospiracaea and Ruminococcaea. As discussed above for glycolysis, the lack of ubiquitous detection most likely relates to differential abundance of the phyla within the rumen microbiome or to a lack of sufficient annotated sequence data. Succinate dehydrogenase was found present in all three cows. This enzyme catalyses the synthesis of fumarate from succinate, which is required for both aerobic and anaerobic growth. While the succinate pathway, acrylate pathway and the propanediol pathway all produce propionate, the succinate pathway is the one most commonly used by bacteria^48^. Bacteriodetes was found to be the most prevalent phylum representative of this pathway in this study, supporting previous observations of this group utilising the succinate pathway via methylmalonyl CoA^49^.

Glutamate dehydrogenase was amongst the most abundant protein functions identified from our dataset, dominated by representatives from the Bacteriodetes family Prevotellacea. Glutamate dehydrogenase is important for the catalysis of α-ketogluterate to glutamate and in enabling assimilation of ammonia within the rumen and microbial protein synthesis^50^. The efficiency by which nitrogen is captured is important for the creation of readily available and utilisable sources of energy for microbial protein synthesis^51^. Nitrogen fixing species within the rumen have previously been described by Hungate^2^ using traditional culture techniques. Although the reduction of gaseous nitrogen to ammonia occurs in lower abundances than some other processes, our meta-proteomic analysis showed an abundance of nitrogen fixing proteins in the rumen.

In conclusion, the most abundant proteins identified from this meta-proteomics based study were determined as those involved in known major metabolic pathways within the rumen. While these were distributed across several major phyla, the activity of the Bacteriodetes dominated the metaproteome, within which the family Prevotellaecae was most abundant. This is unsurprising as Prevotella are typically associated with microbial proteolytic activity in the rumen^52^ and recent analyses indicate a role in early colonisation associated with fibre degradation^11,44,53^. The functional analysis of the roles of bacterial phyla within the rumen have demonstrated the potential for diversified niches and although some overlap has been determined, differentiation between metabolic pathways can be observed. This correlates with the investigation by Rubino *et al*.^11^ where significant differences were observed in key metabolic processes, maintaining niche specialisation.

This study has highlighted that while in general the most abundant proteins of the rumen appear to be associated with the most highly expressed genes from published rumen microbiome data, the relationship between individual proteins and relative transcript abundance is not straightforward. Although the transcriptomic data is informative there is still a potential mismatch with translated data. Therefore, the continued development of methods which provide greater sensitivity, detailed annotation and a more in depth analysis from the meta-proteome have the potential to reveal wider distribution of functionality amongst the rumen meta-proteome, enabling greater understanding of the resilience: redundancy balance within this ecosystem.

## Materials and Methods

### Collection of rumen fluid

Rumen fluid was obtained from three fistulated non-lactating Holstein-Friesian dairy cows from the same herd. All experimentation using animals was conducted under the Animals in Experimentation Act 1986 (UK Home Office Licence number 40-3579) under the authority of EU directive 2010/63/EU. The cows were under the same feeding regime where all had free access to a perennial ryegrass pasture diet prior to commencement of the experiment. Samples were collected after overnight fasting. A rumen sample was taken from each animal and up to one litre of rumen fluid was squeezed through gauze to filter out fibrous material. Each sample was maintained at 39 °C until sample preparation and for no longer than 2 hours.

### Protein extraction and visualisation

Processed rumen fluid from each animal was split into 2 × 500 ml aliquots and centrifuged at 4 °C for 30 minutes at 8000 × g to remove fibrous matter. The supernatant was aspirated from the pelleted material before the supernatant was centrifuged at 100,000 × g for 30 minutes at 4 °C. The resulting pellet was washed×3 with 0.9% NaCl solution^12^ and then further separated from potentially interfering contaminants such as humic substances using 40% Percoll solution (Sigma) layered underneath the pellet and centrifuged at 10, 000 × g (with slow acceleration and deceleration) for 30 min at 4 °C. The pellet was subsequently re-suspended in 0.2% PBS solution to wash the pellet and then centrifuged for 10 min at 12 000 × g at 4 °C. The supernatant was removed and the resulting pellet was resuspended in 5 ml of lysis buffer (7 M urea, 2 M thiourea, 4% (w/v) CHAPS, 10 mM Tris, 1 mM EDTA, 50 mM dithiothreitol (DTT), protease inhibitors (1 tablet/10mls, Roche, UK) and sonicated on ice for 8 × 30 second busts. The samples were then precipitated with 20% TCA, 1% phosphotungstic acid, 0.2% DTT in acetone, overnight at −20 °C followed by x3 30 minute washes at −20 °C in acetone containing 0.2% DTT. The resulting pellets were air dried and re-suspended in rehydration buffer (8 M urea, 2 M thiourea, 4% CHAPS, 13 mM DTT, for 2 hours at room temperature. Samples were then centrifuged for 45 min at 13, 000 × g. Quantification of protein content in prepared samples was conducted by the method of Bradford^54^. Proteins were separated on a one-dimensional SDS-PAGE gel (12.5%T, 3.3%C) and bands were visualised using Coomassie blue staining.

### Protein identification and database searching

Bands were manually excised and subjected to an in-gel tryptic digest according to the method of Shevchenko *et al*.^55^, with slight modification^56^. In brief, sequencing grade modified trypsin (Promega, UK) was diluted to 6.25 ng/µl in 25 mM NH_4_HCO_3_ and incubated at 37 °C overnight. Digested protein samples were resuspended in 20 µl 0.1% formic acid for LC MS/MS analysis on an Agilent 6550 iFunnel Q-TOF mass spectrometer with a Dual AJS ESI source coupled to an 1290 series HPLC system (Agilent, Cheshire, UK). A 2.1 × 50 mm 1.8 micron Zorbax Eclipse Plus C18 column was used. 10 µL of sample was injected for analysis. Liquid chromatography was performed at a flow of 0.1 mL/minute with a piece-linear gradient using water with 0.1% v/v formic acid (A) and acetonitrile with 0.1% v/v formic acid (B)(0 to 3% B over 2 minutes, 3 to 40% B over 7 minutes, 40 to 100% B over 1 minutes, hold at 100% B for 1 minute). Ions were generated using a Dual AJS ESI source. Tandem mass spectrometry was performed in AutoMS/MS mode in the 300 to 1700 range, at a rate of 0.6 spectra per second, performing MS2 on the 5 most intense ions in the precursor scan. Masses were excluded for 0.1 min after MS2 was performed. Reference mass locking was used for internal calibration using the mass of 922.009798 Da. Resulting ESI MS/MS spectra was analysed using Mass Hunter Qualitative Analysis software (Agilent, U.K), which was then processed using an in-house MASCOT server for peptide identification with the significance threshold set at 0.05. Data base searching was conducted against the NCBI database with a limit of 2 missed cleavages with the variable modification set as the oxidation of methionine and the fixed modification as carbamidomethylation of cysteine.

### Comparison of rumen meta-proteome with published genomic datasets of the rumen microbiome

The peptides generated from the rumen meta-proteome were compared to the meta-analysis and comparative study of several rumen 16S rRNA gene based surveys, which examined the culturable fraction of the rumen microbiome^10^. Here we compared the taxonomic diversity observed from the rumen meta-proteome to taxonomic counts obtained from the 16S data. In addition, meta-proteome data was also searched against highly expressed genes according to ranking within a large recently published gene expression rumen microbiome dataset^18^, which consists of 20 rumen samples obtained from members of the same herd. Each sample was sequenced to produce 945 Gb of meta-genomic data and 120 Gb of meta-transcriptomic data. The meta-genomic assembly was produced using Spherical (http://github.com/thh32/Spherical) with Velvet (1.2.10) as the assembler, kmer of 51, subset size of 50 Gb and 6 iterations and aligned to the meta-genomic assembly with Bowtie2 using default settings. For each aligned sample, Samtools idxstats was used, with default settings, to identify the number of reads aligning to each contig in the metagenome assembly and an FPKM value was then calculated per contig for each sample. Abundances from the meta-proteome dataset were then compared to those matching from the meta-transcriptome dataset.

To determine meta-proteome matches to the Hungate 1000 genomic database, the Hungate 1000 genomes (which at point of analysis in 2017 contained the genomes of 406 rumen bacterial accessions) were used as a database against which the predicted peptides from the metaproteome were aligned using DIAMOND (v0.7.9). A minimum bit score of 40 was used as a cut-off for homologous matches. The output was then filtered by overlap using MGKIT to prevent multiple proteins hitting the same genomic region.

### Data availability

The datasets analysed during the study are available from the corresponding author on reasonable request.

## Electronic supplementary material

Supplementary information
