# Supplementary material for: Meta-proteomics of rumen microbiota indicates niche compartmentalisation and functional dominance in a limited number of metabolic pathways between abundant bacteria
Source: Sci Rep. 2018 Jul 12;8:10504. doi: 10.1038/s41598-018-28827-7 (PMC6043501; doi:10.1038/s41598-018-28827-7)
Supplement: Supplementary file 1 — Supplementary information [file 41598_2018_28827_MOESM1_ESM.pdf]

Meta-proteomics of rumen microbiota indicates niche compartmentalisation and functional dominance in a limited number of metabolic pathways between abundant bacteria.

Authors: Hart, E.H, Creevey, C.J., Hitch, T. and Kingston-Smith A.H.

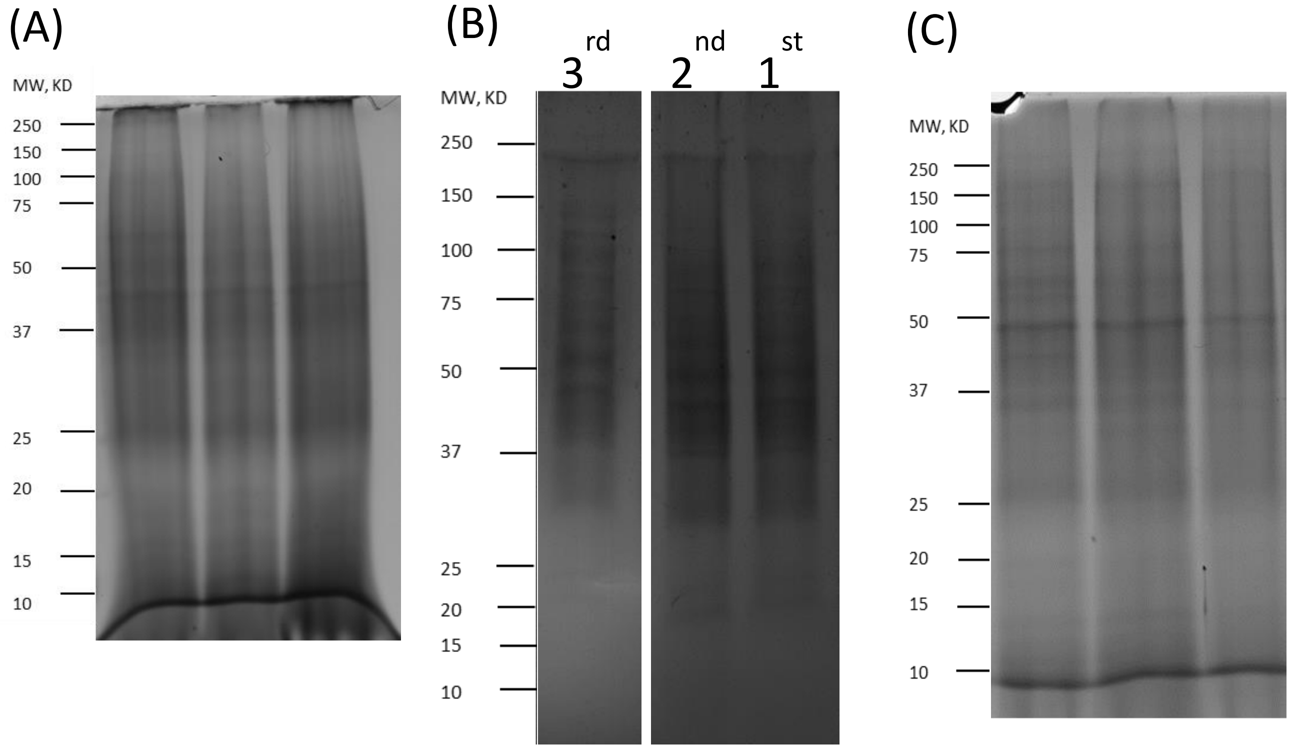

Supplementary Fig S1: Use of NaCl and Percoll prior to SDS PAGE separation of bacterial proteins collected from the rumen. Images show Coomassie blue staining of (A) three independent replicate lysates of bacterial pellets, (B) representative image showing effect of serial washing of the protein pellet with 0.9% NaCl and (C) further separation by centrifugation through 40% Percoll (three independent replicates shown). Protein was loaded at 10 µg per lane in each case.

Supplementary Table S1. Additional information for table 1 in main text. Showing GI and GO numbers for the most abundant protein families and their respective biological processes, molecular function and cellular location

GI number	Uniprot ID/NCBI ref	Name	% abundance			GO Identifier	Biological Process	Molecular function	Cellular location
			Cow 1	Cow 2	Cow 3				
523985044	R5CN47	Elongation factor Tu	45	7	32	GO:006414	Protein biosynthesis	Elongation factor	Cytoplasm
653245519	WP_027451058	Glyceraldehyde-3-phosphte dehydrogenase	4	18	12	GO:0047100	Glycolysis	Oxidoreductase	Cytoplasm
502830691	D5EUS1	50s Ribosomal protein	5	7	12	GO:0006412	Transcription	Ribonucleoprotein	Cytosol
739031180	WP_036912309	30s Ribosomal protein S1	3	3	9	GO:0000028	Translation	RNA binding	Cytoplasm
653244709	WP_027450262	Glutamate dehydrogenase	7	0	7	GO:0004354	Glutamate biosynthetic process	Protein binding	Cytoplasm
158438446	A8RTE3	Hypothetical protein	1	2	4	GO:0005996	fucose metabolic process	L-fucose isomerase activity	Cytoplasm
502830520	D5EU41	Triose-phosphate isomerase	0	3	2	GO:0006094	Glycolysis	Isomerase	Cytoplasm
148071	P0AA25	Thioredoxin	1	2	2	GO:0000103	Electron transport	Oxidoreductase activity	Cytosol
492438964	F9DQI6	Phosphoglycerate kinase	5	1	2	GO:0006096	Glycolysis	kinase	Cytoplasm
495415362	WP_008140060	Succinate dehydrogenase	1	2	2	GO:0000104	Electron transport	Oxidoreductase	Membrane
502830346	Q9HIMM5	Nitrogen fixing protein	1	2	2	GO:0006879	[2Fe-25]cluster assembly	Binding	Cytoplasm
653244953	WP_027450503	Pyruvate phosphate dikinase	8	10	1	GO:0050242	ATP binding	Kinase	Cytosol
395398505	I6XP12	Transkeletolase	1	2	1	GO:0008152	Pentose-phosphate subunit	Transferase	Cytosol
502829801	D5EVD2	Molecular chaperone Dnak	1	1	1	GO:0006457	DNA replication	Chaperone	Cytoplasm
652835513	WP_027118440	Phosphoenolpyruvate carboxykinase (ATP)	0	1	1	GO:0004612	Gluconeogenesis	Decarboxylase	Cytoplasm
400375082	EJP279921	Ribosomal protein	1	1	1	GO:0000027	Transcription	Ribonucleoprotein	Cytosol
490479181	D1W6B4	Pyruvate ferredoxin (flavodoxin) oxidoreductase	1	1	1	GO:0022900	Electron transport, Nitrogen fixation	Oxidoreductase	Cytoplasm
655547847	WP_028911020	Fucose isomerase	1	1	1	GO:0008736	Carbohydrate metabolism	Isomerase	Cytoplasm

Supplementary Table S1 (cont)

GI number	Uniprot ID/NCBI ref	Name	% abundance			GO Identifier	Biological Process	Molecular function	Cellular location
			Cow 1	Cow 2	Cow 3				
386375786	I1YTE2	DNA-directed RNA polymerase, alpha subunit	0	1	1	GO:0006351	Transcription	DNA binding	Nucleus
502829708	WP_013064684	Rubrerythrin	1	1	1	GO:0005506	Electron transport	Oxidoreductase activity	Cytoplasm
502828136	D5ETII0	Methymalonyl-CoA mutase	0	1	1	GO:0008152	Metabolic process	Isomerase	
739028499	WP_036909675	Energy transducer TonB	1	1	1	GO:0006810	Transport	Energy transducer activity	Membrane
502830505	D5ERZ6	Phosphoglucomutase	1	1	1	GO:0005975	Carbohydrate metabolism	Isomerase	Cytoplasm
502830128	D5EUN0	Fructose-bisphosphate aldolase	0	1	1	GO:0030388	Glycolysis	Fructose-bisphosphate aldolase activity	Membrane
270332477	D1PZ29	Pyruvate synthase	0	1	1	GO:0022900	Electron transport	Oxidoreductase	Cytoplasm
502828116	D5ESB3	ATP synthase subunit beta	2	1	1	GO:0015991	ATP synthesis	Hydrolase	Membrane
